# *GNE* genotype explains 20% of phenotypic variability in GNE myopathy

**DOI:** 10.1212/NXG.0000000000000308

**Published:** 2019-02-01

**Authors:** Oksana Pogoryelova, Ian J. Wilson, Hank Mansbach, Zohar Argov, Ichizo Nishino, Hanns Lochmüller

**Affiliations:** From the Institute of Genetic Medicine (O.P., I.J.W.), Newcastle University, Newcastle upon Tyne, United Kingdom; Ultragenyx Pharmaceutical (H.M.), CA; Department of Neurology (Z.A.), Hadassah-Hebrew University Medical Center, Jerusalem, Israel; Department of Neuromuscular Research (I.N.), National Institute of Neuroscience, National Center of Neurology and Psychiatry, Tokyo, Japan; Department of Neuropediatrics and Muscle Disorders (H.L.), Medical Center—University of Freiburg, Faculty of Medicine, Freiburg, Germany; Centro Nacional de Análisis Genómico (CNAG-CRG) (H.L.), Center for Genomic Regulation, Barcelona Institute of Science and Technology (BIST), Barcelona, Catalonia, Spain; and Children's Hospital of Eastern Ontario Research Institute (H.L.), University of Ottawa, Ottawa, Canada and Division of Neurology, Department of Medicine, The Ottawa Hospital, Ottawa, Canada.

## Abstract

**Objective:**

To test the hypothesis that common *GNE* mutations influence disease severity; using statistical analysis of patient cohorts from different countries.

**Methods:**

Systematic literature review identified 11 articles reporting 759 patients. GNE registry data were used as a second data set. The relative contributions of the *GNE* mutations, homozygosity, and country to the age at onset were explored using linear modeling, and relative importance measures were calculated. The rate of ambulation loss for *GNE* mutations, homozygosity, country, and age at onset was analyzed using Cox proportional hazards models.

**Results:**

A spectrum of symptoms and large variability of age at onset and nonambulatory status was observed within families and cohorts. We estimated that 20% of variability is explained by *GNE* mutations. Individuals harboring p.Asp207Val have an expected age at onset 8.0 (s.e1.0) years later than those without and probability of continued ambulation at age 40 of 0.98 (95% confidence interval [CI] 0.96–1). In contrast, p.Leu539Ser results in onset on average 7.2 (s.e.2.7) years earlier than those without this mutation, and p.Val603Leu has a probability of continued ambulance of 0.61 (95% CI 0.50–0.74) at age 40, but has a nonsignificant effect on age at onset.

**Conclusions:**

GNE myopathy severity significantly varies in all cohorts, with 20% of variability explained by the *GNE* mutation. Atypical symptoms and clinical presentation suggest that physical and instrumental examination should include additional clinical tests. Proven and measurable effect of *GNE* mutations on the disease severity should be factored in patient management and clinical research study for a better data interpretation.

GNE myopathy is an autosomal recessive rare distal myopathy. It is a slow progressing disease caused by mutations in the *GNE* gene, which affects sialic acid synthesis. Clinical course varies from mild to severely debilitating forms leading to end-stage skeletal muscle weakness. The genetic basis for GNE myopathy was established in 2001;^1^ more than 180 mutations are currently known, among them several founder or recurrent mutations.^2–7^ Wide phenotypic variability is described in the literature, even within families carrying the same mutation. Some unique features of GNE myopathy are observed only in certain populations.^8,9^ Population-based studies suggest that *GNE* genotype may influence predisposition to a faster or slower progression of the disease, but were often not large enough to reach statistical significance.^5,6,10^ Molecular mechanisms underlying muscle weakness have been studied in cell and enzyme studies, suggesting that different missense mutations may affect enzymatic activity to a different extent. Primary muscle cells with *GNE* mutations showed a significant reduction in sialic acid levels,^11^ suggesting that this might be the cause of the muscle weakness. A defined link between *GNE* genotype and phenotype would have a major impact on the clinical trial planning and routine disease management and care. The aim of the study is to explore cohort-based studies to test the hypothesis that *GNE* genotype influences GNE myopathy disease severity.

## Methods

### Literature review

PubMed database search was conducted on 12 May 2017 using search words “GNE myopathy,” “HIBM,” “quadriceps sparing myopathy,” “distal myopathy with rimmed vacuoles.” In total, 494 records were identified. Duplicated records, single case reports, and scientific publication based on molecular studies, cells, and animal models were eliminated. Other records were checked for the following: cohort size ≥15 patients, availability of demographic and clinical description, and description of the *GNE* mutations in the cohort. Eleven articles were selected for analysis (total 759 cases, table 1). Seven articles contained per patient data (UK, India, Bulgaria, Iran, China 1, China 2, and Japan 3) and 4 articles (Israel, Japan 1, Japan 2, and Japan 4) provided cohort summary information. In total, n = 360 patients had individual level data, n = 342 with defined age at onset, and n = 247 with known ambulation status.

**Table 1:**
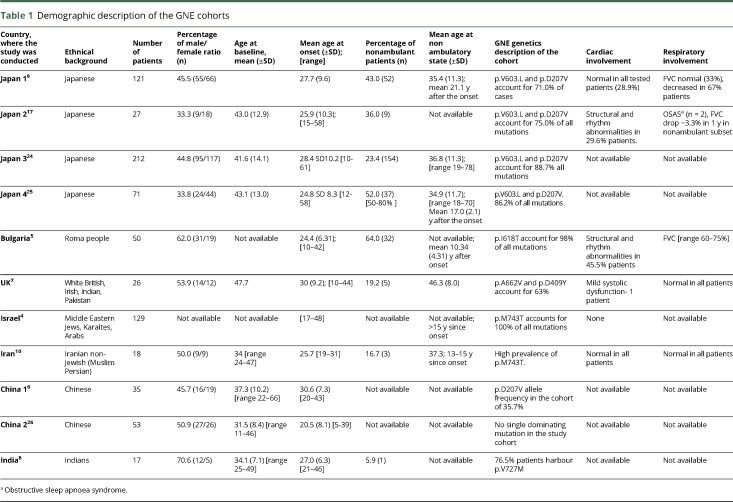
Demographic description of the GNE cohorts

### Registry data

GNE myopathy registry (previously described^12^) was used as another independent data set. The registry data were interrogated for number of patients per country (subset A) and for patients with homozygous mutations regardless of their country of residence (subset B). Six countries were presented by ≥14 patients (total n = 135). Four *GNE* mutations were present in the homozygous state ≥3 patients (total n = 31). The study received approval from Newcastle and North Tyneside 1 Research Ethics Committee, reference number 13/NE/0123. Informed consent was obtained from all patients participating in the registry.

### Statistical modeling of age at onset

Individuals with known genotype and known age at onset were selected for this analysis (n = 342). Those variants with at least 10 observations in the data set were used for further analysis. Ten mutations (table 2) were 77% of the total changes in the data. Genetic explanatory variables were constructed for each mutation under a dominance model, where the variable is coded for the presence/absence of a mutation and allelic dosage model that gives the allele count (0, 1, or 2) for the 10 changes. Other explanatory variables used were the geographic location of the study and whether an individual was homozygous. A series of linear models were fitted for the age at onset regressed against all combinations of location and/or homozygosity and modeling the genetic contribution as either allelic dose or dominance models using the R statistical package.^13^

**Table 2:**
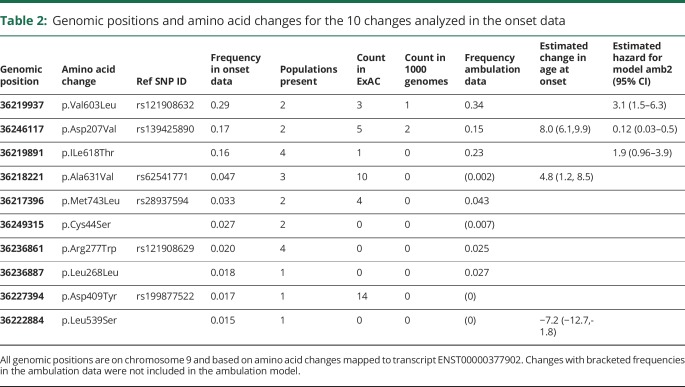
Genomic positions and amino acid changes for the 10 changes analyzed in the onset data

Selection of the better fitting models was done using the stepwise regression with model choice made using the Akaike Information Criterion (AIC).^14^ The relative importance of the different factors in explaining variation in the age at onset was assessed using the relaimpo package within R.^15^ The best fitting models were then used to predict the age at onset for individuals in the GNE registry.

### Age of ambulation loss

Individuals in the onset data that had information about ambulation status, either continued ambulation at assessment or the age at loss of ambulation (n = 220), were used for analysis of ambulation status. Mutations with more than 10 observations in the data set were used for further analysis (n = 6 mutations, table 2). Cox proportional hazards models^16^ were used to evaluate the relationship of continued ambulation to the genotype data, as described above, the study, and the age at onset. Initially univariate survival models were fitted and a threshold *p* value of 0.1 was used to select those variables to consider in a multivariate analysis. A multivariate model was chosen from the best combination of significant univariate models. Choices between non-nested models were made using the AIC. Model fit was assessed using survival concordance for registry individuals.

### Data availability statement

The study analyzes published data. Anonymized GNE registry data can be shared upon request to the GNE Registry Steering Committee.

## Results

### Age at onset

Eleven cohorts were selected for analysis. Four publications from Japan met the inclusion criteria and may have overlapping patients. Two publications from China may have overlapping patients, but this is unlikely. The overlap in patient populations between those cohorts cannot be determined; therefore, they were analyzed as individual patient cohorts. Bulgaria, UK, Israel, Iran, and India are presented by a single publication (table 1).

**Figure 1:**
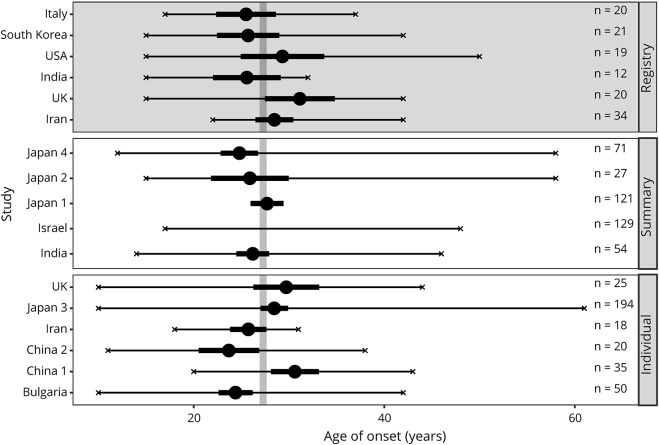
Forest plot showing data overview by onset on a cohort level Studies are grouped by source, individual level data (bottom panel), summary statistics form articles (middle panel), and by country from the registry (top panel with dark background). Means are given by large circles, the confidence intervals by thick black lines, and the minimum and maximum values by x. Sample sizes for each study are indicated to the right. The overall mean (calculated from individual level data) is shown by the vertical grey line. Student-*t* confidence intervals were calculated for registry countries and individual studies. Confidence intervals for summary studies were taken from the articles.

Number of patients in populations varied between 17 (India) and 212 (Japan). Average age at baseline varied between 31.5 years (China) and 47.7 years (UK). The earliest onset at 10 years was detected in Japan, UK, and Bulgaria, while the latest onset was observed at 61 years in Japan, giving Japan the largest variability of age at onset (figure 1).

**Figure 2:**
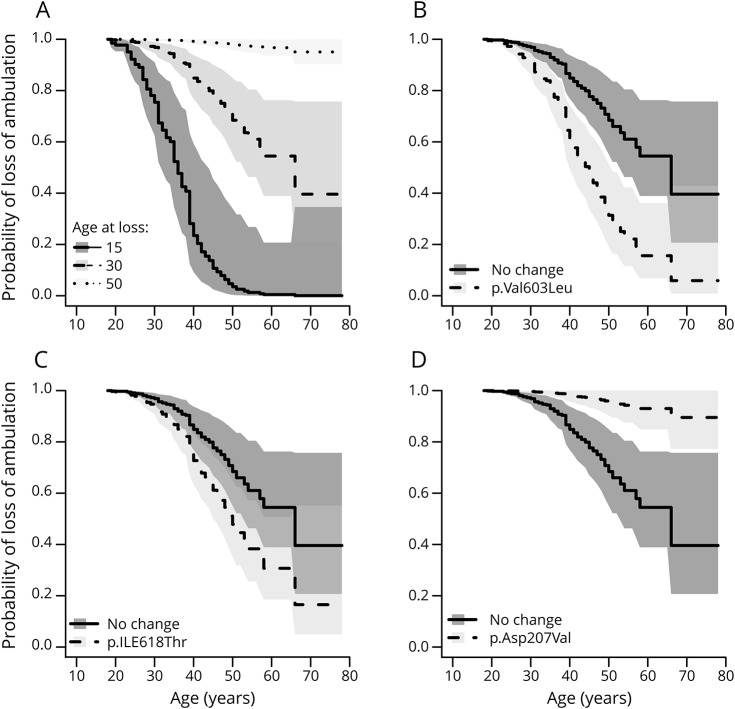
Plots of probability of loss of ambulation with age for Cox-proportional hazard models (A) shows fitted values for model 2 with age, for an individual without p.Val603Leu, p.Ile618Thr, or p.Asp207Val. (B, C and D) give expected curves for an individual with and without the given mutation, with shaded areas indicating 90% confidence intervals.

Linear models were fitted to the age at onset on those individuals with information about genotype and age at onset (n = 341). Genetic variables provided a better fit to the data than location and homozygosity (maximum adjusted r^2^ was 0.08 without individual mutation explanatory variables). Models using allelic dose models were marginally worse than dominance models. Statistically significant mutations were p.Asp207Val (increase in age at onset of 8 years *p* = 9e-16), p.Ala631Val (increase of 4.8 years, *p* = 0.002), and p.Leu539Ser (decrease of 7.2 years, *p* = 0.008). Confidence intervals are given in table 2. The proportion of variability explained by these 3 genetic factors was estimated to be 18.8%, using the reliampo package. For univariate models, the age at onset was a highly significant predictor of continued ambulance (*p* < 2e-16), along with individual homozygosity (*p* = 7e-16), and the mutations, p.Val603Leu (*p* = 0.007), p.Asp207Val (*p* = 0.0002), and p.ILe618Thr (*p* = 3.4e-14). In a multivariate model using all variables homozygosity was only marginally significant (*p* > 0.01). Age at onset and dominance for the 3 mutations — the change in hazard for the 3 mutations — was used in the final model (table 2). The effect of the age at onset and the 3 mutations (figure 2). We can show the effects of the 3 mutations by comparing mutations to an individual with onset at age 30 and without any of the 3 mutations who has a probability of 0.85 (95% confidence interval [CI] 0.77–0.94) of continued ambulance at age 40.

### Age of ambulation

Lowest proportion of nonambulant patients was described in India (5.9%) and the highest in Bulgaria (64.0%), resulting in almost every third GNE patient in the world being nonambulant (32.5%). Authors, of the selected manuscripts, described reaching nonambulatory status in 2 ways: (1) Age at nonambulatory status (5 publications) and duration (i.e., years) since the onset to nonambulatory status (3 publications). The former shows that patients reach nonambulatory status at an average 38.1 years old, with over a decade (11.4 years) difference between “Japan 4” and UK cohorts. The latter shows that the progress since onset to the wheelchair bound state can take between 10.3 and 21.1 years with an average of 16.2 years.

A mutation at p.Val603Leu gives a probability of ambulance at age 40 of 0.61 (95% CI 0.49–0.74), a mutation at p.IleThr gives 0.73 (95% CI 0.163–0.83), and a mutation at p.Asp207Val has a probability of continued ambulance of 0.98 (95% CI 0.96–1). When considering only the Japanese cohort, we confirm that the presence of p.Asp207Val is associated with a much milder phenotype (probability of ambulance at age 40 is 0.98 95% CI [0.94–1.0]), compared to individuals with no copies probability 0.58 [0.45, 0.73]. Homozygotes of p.Val603Leu have a much more severe phenotype with probability of continued ambulance of 0.39 95% CI [0.24–0.61] compared to a heterozygote, probability 0.90 95% CI [0.80–1.0].

### Genotypes

Founder or high-prevalence mutations were significantly prevalent in 9 cohorts. In 2 populations (Israel and Bulgaria), founder mutation appears in nearly all patients in homozygous state. Japan and UK showed 2 mutations dominating the cohorts. China 1 and China 2 cohorts showed a different spectrum of mutations with one dominating mutation in former study and multiple mutations without clear dominance in the latter.

### Cardiorespiratory function

It is considered that GNE myopathy does not increase the risk of cardiomyopathy and respiratory failure. However, a 1 yearlong natural history study conducted in Japan showed that forced vital capacity (FVC) declines in nonambulatory patients.^17^ Description of the respiratory function was available in 5 of 10 studies. UK and Iran described it as “normal in all patients.” FVC was either normal or mildly to moderately decreased in all other tested patients. No severe decline in FVC was reported. Cardiomyopathy was not reported; nevertheless, some structural and rhythm abnormalities were present in 29.6 and 45.5% of patients from “Japan 2” and Bulgaria, respectively. Reported abnormalities included borderline reduction in ejection fraction and impaired relaxation (in patients who had concomitant diseases such as diabetes or hypertension). ECG in a few patients showed evidence of abnormal repolarization and left ventricular hypertrophy. Rhythm abnormalities such as extrasystolic arrhythmia and borderline nonspecific intraventricular delay were observed on a few occasions. Sudden death of unknown cause was reported in 4 nonambulant patients without a family history of cardiac rhythm abnormalities.^5^

### Atypical features

Muscle biopsy appearance without rimmed vacuoles was observed in the UK and Japan and is likely to be linked to the site of the biopsy (i.e., quadriceps as the least affected muscle) or early stage of the disease. Thrombocytopenia was reported in 2 Japanese cohorts (“Japan 1” and “Japan 2”). A high rate of an early onset presentation (<20 years) was reported in “Japan 4” cohort. Asymmetrical weakness was evident in the UK and both Chinese cohorts. Quadriceps sparing, almost a pathognomonic feature of GNE myopathy, was absent in a small number of patients from Israel, China 1, and China 2 cohorts. Facial and neck weakness and limb-girdle muscle weakness at early stages of the disease were observed in China and Israel.

### Analysis of the registry cohort

Age at onset and nonambulatory status were compared between 6 country-specific cohorts, with n ≥ 14 patients. Baseline data are presented in table 3. Using the model and parameter values estimated in from section 6 to predict onset times for the registry data (n = 126) was not fully successful, with an r^2^ of 0.04 between the self-reported onset values in the registry data and the onset values predicted from this model based on the registry genetic data. So, there is a relationship between estimated onset time and predicted time, but this is not as strong as in the meta data set.

**Table 3:**
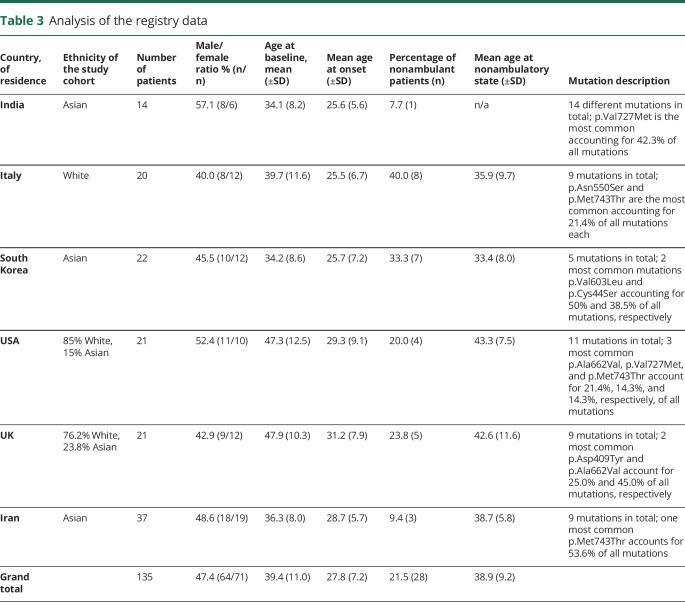
Analysis of the registry data

Registry data with ambulance data had a sample size of 113. Survival concordance for registry individuals was calculated using the parameters from the best fit survival model. This works on pairs of individuals and averages how often the individual with the lowest ambulance time also has the highest risk score. This is then averaged over all pairs of individuals. Hence, perfect matches would have a concordance of 1 and a model that was random would have a concordance of 0.5. The concordance for the registry data was 0.78, which shows good agreement.

Registry data were also interrogated to identify homozygous patients irrespectively of their country of residence. Four subsets with the number of patients ≥4 were analyzed. Because of a small number of available data, we present it only for descriptive analysis. Baseline demographic data, genotype, and clinical parameters are presented in table 3.

## Discussion

Disease severity and variability in its presentation are factors to consider in the disease management and planning clinical research. Patients are keen to know their prognosis and likely trajectory of progression to make personal choices. Rare disease status leads to scarcity of the data and availability of biological samples to study. Therefore, an integrated analysis of the available data provides an additional insight in understanding the correlation between *GNE* genotype and phenotype.

Cohort-based studies provided country-specific data. Analysis of these cohorts showed the presence of outliers with an early or late onset of the first symptoms in every cohort, which is not explained by a particular mutation. We find that almost 20% of the variability in age at onset is due to the *GNE* genotype, with the change to p.Asp207Val being associated with on average an 8-year increase in age at onset. In contrast, p.Leu539Ser results in onset on average 7.2 years earlier than those without this mutation.

*Escherichia coli* and insect cell disease models showed that epimerase and kinase enzymatic activity significantly varied among selected mutations.^18^ Slow progression of the disease may partially be explained by mitochondrial alterations.^19^ However, the correlation between the mutations and level of membrane sialylation is not consistent, which may indicate that involvement of other factors affecting the cellular and clinical phenotype.^20^ An indirect marker of disease severity–age of nonambulation status, has a marked variability.^12^ The differences between age at nonambulatory status were associated with the age at onset and 3 founder mutations. While it is difficult to disentangle population effects from genetic effects, special fitting models for nonambulatory age were better fitted to genetic data than to population source, and these models showed good concordance with those used to predict continued ambulance in the GNE registry. Significant difference between Japan, Iran, and Bulgaria in age at nonambulatory status and proportion of nonambulant patients in the cohorts also point to the fact that the speed at which the disease progresses may be attributable to the founder mutations highly prevalent in those populations. When comparisons are made only with homozygous Japanese populations, the differences in survival curves almost disappear. Roma people founder mutation (p.ILe618Thr) shows no evidence of association with the age at onset, but is associated with a lower age of loss of ambulation. The mutation p.Leu539Ser, only seen in the Chinese data, is associated with an onset 7.2 years earlier but is not seen in the ambulation data, and we cannot make any inferences about its impact on ambulation. As these mutations are not shared with other study populations, it is difficult to extract the effects of genotype. The differences in health care approaches can also potentially play a role in preserving ambulation.

Cohorts with a higher number of nonambulant patients also have a higher number of patients with compromised respiratory function. This confirms previous observation on a multinational level that respiratory function is normally preserved until the very nonambulatory stages of the disease. We also did not observe a correlation between cardiomyopathy and GNE myopathy but a number of mild-to-moderate structural and rhythm heart abnormalities were observed.

Rare symptoms found only in one country (e.g., thrombocytopenia or Beevor sign) could be attributable to the described ethnic groups only or to observational bias due to the differences in medical practices across the world. A new *GNE* mutation has been found to cause a congenital macrothrombocytopenia without noticeable muscle weakness to date.^21^ It supports the observation that platelet count may be compromised in GNE myopathy and therefore it may be recommended to include platelet count test in the routine management of the disease.

Spectrum of the disease presentation, severity, and specific features is important in planning clinical trials for adequate patient stratification and reduced bias due to known covariances. N-acetylneuraminic acid extended release (Ace-ER) phase 2 and 3 clinical trials showed different results, where phase 2 showed that Ace-ER stabilizes the disease progression and causes statistical significant difference between treatment and placebo groups.^22^ However, phase 3 showed no benefit of Ace-ER on muscle strength.^23^ We speculate that difference in the results between 2 studies could partially be accounted for differences in the study cohorts. In phase 2 trial, sites were located in 2 countries, the United States and Israel, where p.Met743Thr is most common. Phase 3 study enrolled a larger number of patients from 7 countries, with 4 of them being in Europe and one in Canada—where p.Met743Thr is much less prevalent. This genotype difference in the cohorts could have potentially had an impact on the discrepancy in the study results. We therefore suggest that *GNE* mutation should be taken in consideration as a factor for patient stratification in the clinical trials.

This article is a first attempt to look at the genotype—phenotype correlation in GNE myopathy through a systematic review and meta-analysis. The analysis shows that *GNE* genotype has an impact on the phenotype and accounts for 20% of variability in onset of the disease and has a significant influence on reaching nonambulatory status. The analysis indicated that there are other disease modifying factors that influence phenotype. Cohort-specific features expand clinical understanding of the disease and warrant a closer look at the platelet count in GNE patients across the world. Better understanding of the disease progression based on the genotype is important for counselling and management of GNE patients. Potential impact of genotype on the disease progression is also important for clinical trial design and patient stratification. We acknowledge study limitations, such as sample size, and factors (e.g., differences in environment, diet, and health care systems) that can potentially influence the analysis. Therefore, a replication of the analysis on a larger and different cohort would have to be conducted in the future.

## Publication history

Received by *Neurology* September 7, 2018. Accepted in final form December 20, 2018.

## Glossary

CI: confidence interval
Ace-ER: N-acetylneuraminic acid extended release
AIC: Akaike Information Criterion

## References

[1] Eisenberg I, Avidan N, Potikha T, et al The UDP-N-acetylglucosamine 2-epimerase/N-acetylmannosamine kinase gene is mutated in recessive hereditary inclusion body myopathy. Nat Genet 2001;29:83–87. 11528398 10.1038/ng718

[2] Celeste FV, Vilboux T, Ciccone C, et al Mutation update for GNE gene variants associated with GNE myopathy. Hum Mutat 2014;35:915–926. 24796702 10.1002/humu.22583 PMC4172345

[3] Nishino I, Noguchi S, Murayama K, et al Distal myopathy with rimmed vacuoles is allelic to hereditary inclusion body myopathy. Neurology 2002;59:1689–1693. 12473753 10.1212/01.wnl.0000041631.28557.c6

[4] Argov Z, Eisenberg I, Grabov-Nardini G, et al Hereditary inclusion body myopathy: the Middle Eastern genetic cluster. Neurology 2003;60:1519–1523. 12743242 10.1212/01.wnl.0000061617.71839.42

[5] Chamova T, Guergueltcheva V, Gospodinova M, et al GNE myopathy in Roma patients homozygous for the p.I618T founder mutation. Neuromuscul Disord 2015;25:713–718. 26231298 10.1016/j.nmd.2015.07.004

[6] Zhao J, Wang Z, Hong D, et al Mutational spectrum and clinical features in 35 unrelated mainland Chinese patients with GNE myopathy. J Neurol Sci 2015;354:21–26. 25986339 10.1016/j.jns.2015.04.028

[7] Chaouch A, Brennan KM, Hudson J, et al Two recurrent mutations are associated with GNE myopathy in the North of Britain. J Neurol Neurosurg Psychiatry 2014;85:1359–1365. 24695763 10.1136/jnnp-2013-306314 PMC6625961

[8] Preethish-Kumar V, Pogoryelova O, Polavarapu K, et al Beevor's sign: a potential clinical marker for GNE myopathy. Eur J Neurol 2016;23:e46–48. 27431025 10.1111/ene.13041

[9] Mori-Yoshimura M, Hayashi YK, Yonemoto N, et al Nationwide patient registry for GNE myopathy in Japan. Orphanet J Rare Dis 2014;9:150. 25303967 10.1186/s13023-014-0150-4 PMC4203883

[10] Haghighi A, Nafissi S, Qurashi A, et al Genetics of GNE myopathy in the non-Jewish Persian population. Eur J Hum Genet 2016;24:243–251. 25966635 10.1038/ejhg.2015.78 PMC4717203

[11] Noguchi S, Keira Y, Murayama K, et al Reduction of UDP-N-acetylglucosamine 2-epimerase/N-acetylmannosamine kinase activity and sialylation in distal myopathy with rimmed vacuoles. J Biol Chem 2004;279:11402–11407. 14707127 10.1074/jbc.M313171200

[12] Pogoryelova O, Cammish P, Mansbach H, et al Phenotypic stratification and genotype-phenotype correlation in a heterogeneous, international cohort of GNE myopathy patients: first report from the GNE Myopathy Disease Monitoring Program, registry portion. Neuromuscul Disord 2018;28:158–168. 29305133 10.1016/j.nmd.2017.11.001 PMC5857291

[13] Team RC. R: A Language and Environment for Statistical Computing. Austria; R Foundation for Statistical Computing Vienna; 2018.

[14] Akaike H A new look at the statistical model identification. IEEE Trans Automatic Control 1974;19:716–723.

[15] Relative UG. Importance for linear regression in R: the package relaimpo. J Stat Softw 2006;17:1–27.

[16] Venables WN, Ripley BD Modern Applied Statistics with S-Plus. Berlin, Germany: Springer-Verlag; 1994.

[17] Mori-Yoshimura M, Oya Y, Yajima H, et al GNE myopathy: a prospective natural history study of disease progression. Neuromuscul Disord 2014;24:380–386. 24656604 10.1016/j.nmd.2014.02.008

[18] Penner J, Mantey LR, Elgavish S, et al Influence of UDP-GlcNAc 2-epimerase/ManNAc kinase mutant proteins on hereditary inclusion body myopathy. Biochemistry 2006;45:2968–2977. 16503651 10.1021/bi0522504

[19] Eisenberg I, Novershtern N, Itzhaki Z, et al Mitochondrial processes are impaired in hereditary inclusion body myopathy. Hum Mol Genet 2008;17:3663–3674. 18723858 10.1093/hmg/ddn261

[20] Salama I, Hinderlich S, Shlomai Z, et al No overall hyposialylation in hereditary inclusion body myopathy myoblasts carrying the homozygous M712T GNE mutation. Biochem Biophys Res Commun 2005;328:221–226. 15670773 10.1016/j.bbrc.2004.12.157

[21] Futterer J, Dalby A, Lowe GC, et al Mutation in GNE is associated with a severe form of congenital thrombocytopenia. Blood 2018;132:1855–1858. 29941673 10.1182/blood-2018-04-847798 PMC6238157

[22] Argov Z, Caraco Y, Lau H, et al Aceneuramic acid extended release administration maintains upper limb muscle strength in a 48-week study of subjects with GNE myopathy: results from a phase 2, randomized, controlled study. J Neuromuscul Dis 2016;3:49–66. 27854209 10.3233/JND-159900 PMC5271423

[23] Lochmüller H, Behin A, Caraco Y, et al A phase 3 randomized study evaluating sialic acid extended-release for GNE myopathy. Neurology Epub 2019 Jan 25. 10.1212/WNL.0000000000006932 PMC6512882 31036580

[24] Cho A, Hayashi YK, Monma K, et al Mutation profile of the GNE gene in Japanese patients with distal myopathy with rimmed vacuoles (GNE myopathy). J Neurol Neurosurg Psychiatry 2014;85:914–917. 24027297 10.1136/jnnp-2013-305587

[25] Mori-Yoshimura M, Monma K, Suzuki N, et al Heterozygous UDP-GlcNAc 2-epimerase and N-acetylmannosamine kinase domain mutations in the GNE gene result in a less severe GNE myopathy phenotype compared to homozygous N-acetylmannosamine kinase domain mutations. J Neurol Sci 2012;318:100–105. 22507750 10.1016/j.jns.2012.03.016

[26] Lu X, Pu C, Huang X, Liu J, Mao Y Distal myopathy with rimmed vacuoles: clinical and muscle morphological characteristics and spectrum of GNE gene mutations in 53 Chinese patients. Neurol Res 2011;33:1025–1031. 22196754 10.1179/1743132811Y.0000000070

